# The challenges of replication

**DOI:** 10.7554/eLife.23693

**Published:** 2017-01-19

**Keywords:** Reproducibility Project: Cancer Biology, replication, reproducibility, methodology, open science, metascience

## Abstract

Interpreting the first results from the Reproducibility Project: Cancer Biology requires a highly nuanced approach.

Reproducibility is a cornerstone of science, and the development of new drugs and medical treatments relies on the results of preclinical research being reproducible. In recent years, however, the validity of published findings in a number of areas of scientific research, including cancer research, have been called into question (Begley and Ellis, 2012; Baker, 2016). One response to these concerns has been the launch of a project to repeat selected experiments from a number of high-profile papers in cancer biology (Morrison, 2014; Errington et al., 2014).

The aim of the Reproducibility Project: Cancer Biology, which is a collaboration between the Center for Open Science and Science Exchange, is two-fold: to provide evidence about reproducibility in preclinical cancer research, and to identify the factors that influence reproducibility more generally.

The project is employing a Registered Report/Replication Study approach to publish its work and results. The basic idea behind this approach is that a Registered Report detailing the proposed experimental designs and protocols for each replication is peer reviewed and published after suitable revisions. Crucially, data collection cannot begin until the Registered Report has been accepted for publication. The results of the experiments are then published as a Replication Study, irrespective of the outcome, but subject to peer review to check that the designs and protocols contained in the Registered Report were followed.

The papers included in the project were all published between 2010 and 2012, and were selected on the basis of search terms (such as cancer, onco* and tumor*). Certain types of papers were excluded (such as clinical trials), as were papers that required specialized samples, techniques or equipment that would be difficult or impossible to obtain. The papers selected for replication were those with the highest citation rates and altmetric scores (see Errington et al., 2014 for a full description of the selection process). There was no suggestion that any paper was or was not likely to be reproducible.

Since the publication of the original studies, published and unpublished results from other labs have suggested that a number of the studies are reproducible, but there is concern that some of them may not be reproducible. The existence of such data did not alter the efforts of the Reproducibility Project to independently assess the reproducibility of the original studies.

For every paper the team performing the replication contacted the corresponding author of the original paper for additional information to help prepare the Registered Report. The corresponding author was also asked to comment on this report during the peer review process and some, but not all, availed of this. It is important to note that only selected experiments (or figures) from the original paper would be repeated, and in some cases these did not include key experiments in the original studies. In other cases the most interesting implications of the original studies were not tested.

It is also important to note that even if all the original studies were reproducible, not all of them would be found to be reproducible, just based on chance. The experiments in the Reproducibility Project are typically powered to have an 80% probability of reproducing something that is true: this means that if we attempt to repeat three experiments from a paper, there is only a ~50% chance that all three experiments will yield significant p values, even if the original study was reproducible. Therefore, we cannot place the bar so high that the replications need to hit a significant p value in every experiment. If a replication reproduces some of the key experiments in the original study, and sees effects that are similar to those seen in the original in other experiments, we need to conclude that it has substantially reproduced the original study.

The original plan was to conduct 50 replications but some had to be dropped for budget reasons, and a small number of Registered Reports did not make it through peer review as reviewers decided that it would not be possible to draw meaningful conclusions from the proposed experiments. The first Registered Reports were published in December 2014 and a total of 29 have been published to date. Areas of concern that emerged during the peer review process included the limited budget for in vivo experiments and, in some cases, the possibility that the scope of the proposed experiments might not be sufficient to adequately explore the reproducibility of the original studies.

The first five Replication Studies have now been published. Two of the studies reproduced important parts of the original papers (Kandela et al., 2017; Aird et al., 2017), and one did not (Mantis et al., 2017). The other two Replication Studies were uninterpretable because the control tumors grew too quickly or too slowly (or exhibited spontaneous regressions) to reliably measure whether the experimental intervention had the predicted effect (Horrigan et al., 2017a; Horrigan et al., 2017b): however, in one of these two cases the original paper (Willingham et al., 2012) has led to clinical trials for anti-CD47 antibody therapy that will provide extensive additional data on the effectiveness of this approach. Three of the Replication Studies are also accompanied by Insight articles (Dang, 2017; Davis, 2017; Sun and Gao, 2017).

Although it is obviously too early to draw any conclusions about the reproducibility of research into cancer biology on the basis of such a limited dataset, some clear messages have emerged. In particular, the experiments reported in the Replication Studies provide one indication of how readily reproducible previously published results are, but they cannot be considered conclusive evidence of the reproducibility, or lack of reproducibility, of any one study. For that, it will be necessary for the scientific community to aggregate results from multiple attempts by multiple groups".

This approach taken by the Reproducibility Project: Cancer Biology is itself an experiment and, again, it is too early to say whether it is working. A potential strength of the approach is that the experiments are performed by disinterested third parties with no vested interest in whether the experiments reproduce or not. However, this is also a potential disadvantage because the contract research laboratories performing the replications may not have the same level of expertise or motivation as the original laboratories.

The first five Replication Studies have also highlighted a potentially serious shortcoming of the Registered Report/Replication Study approach. The practice of specifying in advance precisely which experiments will be done, down to numbers of cells and replicates, is a strength because it avoids the possibility of biasing outcomes by mid-course changes in experiment. However, it has also proved to be a weakness in some cases because it has prevented experiments from being redone in different ways when the results were uninterpretable. This happened in a number of cases where control tumors grew with different kinetics than in the original studies despite attempts to use the same cells, same cell doses and same recipient mice.

An academic laboratory confronted with this situation while making a serious effort to determine whether a result is reproducible would perform the experiments in different ways, with different conditions, to generate clear results and to test whether there is some condition under which the original observation holds. However, restricting the scientists performing the replications to the experimental designs in the Registered Report meant that, in general, they were not able to redo the experiments with different cell doses to achieve more interpretable kinetics. This has been particularly problematic with tumor formation assays in vivo, in which variability is often high and results depend upon the experience of the investigator.

We will publish more Replication Studies over the months ahead and, at the conclusion of the project, a meta-analysis of all the studies (Errington and Nosek, 2017). While we wait for this, it is important not to overinterpret the results. Already it is clear that nuanced interpretations are necessary, not black and white conclusions about which studies reproduced and which did not. It is also clear that this approach to testing reproducibility remains an experiment, with advantages and disadvantages, including the fact that it sometimes yields results that cannot be interpreted.
